# Deep Convolutional Recurrent Model for Automatic Scoring Sleep Stages Based on Single-Lead ECG Signal

**DOI:** 10.3390/diagnostics12051235

**Published:** 2022-05-15

**Authors:** Erdenebayar Urtnasan, Jong-Uk Park, Eun Yeon Joo, Kyoung-Joung Lee

**Affiliations:** 1 Artificial Intelligence Big Data Medical Center, Wonju College of Medicine, Yonsei University, Wonju 26417, Korea; edenbyra@yonsei.ac.kr; 2 Department of Medical Artificial Intelligence, Medical Engineering College, Konyang University, Daejeon 35365, Korea; jupark@konyang.ac.kr; 3 Department of Neurology, Samsung Medical Center, School of Medicine, Sungkyunkwan University, Suwon 16419, Korea; eunyeon.joo@gmail.com; 4 Department of Biomedical Engineering, College of Health Science, Yonsei University, Wonju 26493, Korea

**Keywords:** electrocardiogram, automatic sleep scoring, deep learning, convolutional neural network, recurrent neural network, deep convolutional recurrent network

## Abstract

Background: Sleep stage scoring, which is an essential step in the quantitative analysis of sleep monitoring, relies on human experts and is therefore subjective and time-consuming; thus, an easy and accurate method is needed for the automatic scoring of sleep stages. Methods: In this study, we constructed a deep convolutional recurrent (DCR) model for the automatic scoring of sleep stages based on a raw single-lead electrocardiogram (ECG). The DCR model uses deep convolutional and recurrent neural networks to apply the complex and cyclic rhythms of human sleep. It consists of three convolutional and two recurrent layers and is optimized by dropout and batch normalization. The constructed DCR model was evaluated using multiclass classification, including five-class sleep stages (wake, N1, N2, N3, and rapid eye movement (REM)) and three-class sleep stages (wake, non-REM (NREM), and REM), using a raw single-lead ECG signal. The single-lead ECG signal was collected from 112 subjects in two groups: control (52 subjects) and sleep apnea (60 subjects). The single-lead ECG signal was preprocessed, segmented at a duration of 30 s, and divided into a training set of 89 subjects and test set of 23 subjects. Results: We achieved an overall accuracy of 74.2% for five classes and 86.4% for three classes. Conclusions: These results show the DCR model’s superior performance over those in the previous studies, highlighting that the model can be an alternative tool for sleep monitoring and sleep screening.

## 1. Introduction

Sleep is an important physiological rhythm in humans and occupies a significant portion, about one-third, of a person’s life. It has a complex structure and cyclic rhythm that can define its quality and efficiency. Sleep structure consists of the following stages: wake, stage 1, stage 2, stage 3, stage 4, and rapid eye movement (REM) sleep. Stages 3 and 4 are often combined and called either N3 or slow wave sleep, and stages 1 to 4 are combined to form the non-REM (NREM) stages [1]. During sleep, the brain organizes the learned content, removes toxins, and recharges energy [2]. Abnormal sleep can lead to a number of illnesses such as daytime sleepiness [3], headaches [4], cardiovascular disease [5], decreased cognitive function [6], and decreased immunity [7]. It is also important to have a good daytime in order to prevent any abnormalities when sleeping. The number of people suffering from sleep disorders, including insomnia, sleep fragmentation, and sleep apnea, is increasing; thus, it is necessary to diagnose these problems appropriately through a systematic sleep analysis [8].

Polysomnography (PSG) is the gold standard of diagnostic tools for evaluating sleep structure and sleep fragmentation. For PSG recording, a technician applies the electrodes to the body surface of a patient, who goes to sleep at the sleep center, to measure various bio-signals (e.g., EEG, EOG, ECG, and EMG). Based on the bio-signals obtained from the patient’s PSG, sleep disorders can be objectively diagnosed, along with sleep fragmentation and structure. Among the PSG recordings, EEG and EOG are the initial bio-signals that can help assess sleep stages. Numerous studies have proposed sleep stage scoring methods based on EEG [9,10,11,12].

An electrocardiogram (ECG) is an alternative vital sign for classifying sleep stages in home healthcare. Recently, various studies have proposed methods for the automatic classification of sleep stages using ECG signals. Initially, they extract intermediate signals, such as heart rate (HR), beat-to-beat (RR) interval, and heart rate variability (HRV) signals, from a raw ECG signal. Adnane et al. [13] studied an approach that not only classifies sleep and wake stages, but also calculates sleep efficiency using a detrended fluctuation analysis of HRV. In contrast, Xiao et al. [14] proposed an alternative method for sleep stage classification based on a random forest classifier with 41 comprehensive features of a HRV signal. They classified the sleep stages into three classes: wake, NREM, and REM, using a selection of 21 features. Singh et al. [15] studied a distinction method for REM and NREM sleep staging based on a support vector machine (SVM) classifier using the features of RR interval. The features were extracted from the temporal, spectral, and nonlinear domains, and one spectral and four nonlinear features were chosen for the final selection. Recently, Yücelbaş et al. [16] identified wake, NREM, and REM stages based on morphological and nonlinear feature sets obtained from ECG signals. To do this, they first extracted intermediate vital signs, including HR, HRV, and RR intervals. Then, the number of features was obtained by analyzing them in the time, frequency, and nonlinear domains. Finally, the top features were selected to reduce the number of features for the training random forest classifier; however, all these studies do not find intermediate features or signals (including HR, HRV, and RR intervals from ECG signal) to extract a number of features using a domain transformation or high-dimensional analysis. In addition, they do not select highly discriminative features to reduce the number of features for classifier training.

Deep learning approaches, including deep neural networks (DNNs), convolutional neural networks (CNNs), and long short-term memory (LSTM), have been used in sleep monitoring and sleep stage classification from a single-lead ECG signal. Wei et al. [17] designed a four-layer DNN model for sleep stage classification, such as wake, REM, and NREM; however, they used 11 handcrafted features obtained from ECG signals with a relatively low accuracy of 77.0%. Li et al. [18] classified sleep stages based on a deep CNN model using a single-lead ECG signal. First, they extracted HRV and ECG-derived respiration (EDR) from the ECG to obtain the cardiorespiratory coupling (CRC) data. CRC data were used as inputs to the CNN model to extract the feature sets. Finally, CNN features and some nonlinear features were applied to an SVM classifier for the final selection; however, these studies use a single-lead ECG with intermediate vital signs, including a RR interval, HR, and HRV, to obtain various handcrafted feature sets. In addition, Radha et al. [19] proposed an LSTM-based method, in which 135 features were extracted from the HRV signals for five-class sleep stages [19]. In addition, a CNN model was used as the feature extractor, and the LSTM model was reused through transfer learning; therefore, an easy and accurate method is needed for the automatic scoring of sleep stages based on a raw single-lead ECG signal.

In this study, we construct a deep convolutional recurrent (DCR) model for the automatic scoring of sleep stages based on a raw single-lead ECG signal. The DCR model consists of convolutional and recurrent neural networks that consider the complex structure and cyclic rhythm of human sleep. A single-lead ECG is used without the extraction of any intermediate vital signs, including the RR interval, HR, HRV, and other handcrafted features. The constructed DCR model is trained and evaluated using clinical PSG datasets from normal subjects and patients with obstructive sleep apnea (OSA).

## 2. Materials and Methods

**Figure 1:**
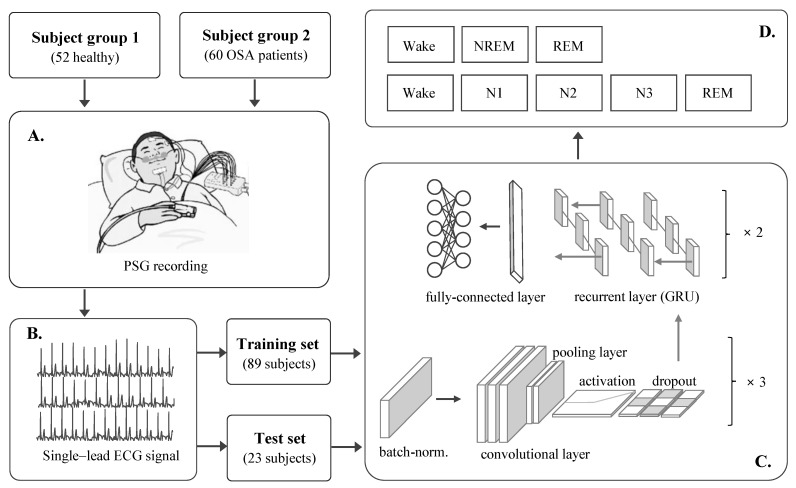
Schematic diagram of this study. (**A**) Study population and PSG study, (**B**) ECG dataset, (**C**) DCR model, and (**D**) target sleep stages.

This study constructs a DCR model for automatic sleep stage scoring based on a raw single-lead ECG signal. The proposed method consists of four main parts: subjects and PSG study, ECG dataset, DCR model, and evaluation, as shown in Figure 1. Each part of the study is explained in detail in the following subsections.

### 2.1. Subjects and PSG Study

We trained and tested the DCR model for automatic sleep stage scoring on a raw single-lead ECG dataset obtained from two different subject groups. The first was the control group, which comprised 52 nocturnal PSG recordings of healthy participants (25 males, 27 females). The second group consisted of 60 subjects with OSA, including mild (15 males, 7 females) and moderate (31 males, 7 females) groups, as shown in Table 1.

All PSG recordings were measured using a polygraphic amplifier (N7000, Embla, Iceland) with an average duration of 7.4 h at Samsung Medical Center, Seoul, Korea. PSG data were composed of EEG, EOG, EMG, ECG, chest and abdomen respiration, airflow, oxygen saturation, and snoring. Signals were recorded at a sampling rate of 200 Hz and were segmented in units of 30 s. Sleep stages were labeled according to the criteria of the American Association of Sleep Medicine [20].

The PSG study was approved by the Institutional Review Board (IRB No. 2012-01-063) of Samsung Medical Center. Patients with severe OSA and cardiovascular disease were excluded from this study.

### 2.2. ECG Dataset

The ECG signal was collected using a single-lead transducer from each subject group, and the episodes were sequentially sampled with 6000 samples per episode. Normalization was applied for preprocessing the ECG signal. A total of 100,395 episodes were obtained after combining 112 healthy subjects (control group) and patients (OSA group) into the entire ECG dataset. Datasets were built from randomly selected subjects from each group for the training and testing of the constructed DCR model. The training set consisted of 80,316 episodes from 89 subjects, whereas the test set comprised 20,079 episodes from 23 subjects.

**Figure 2:**
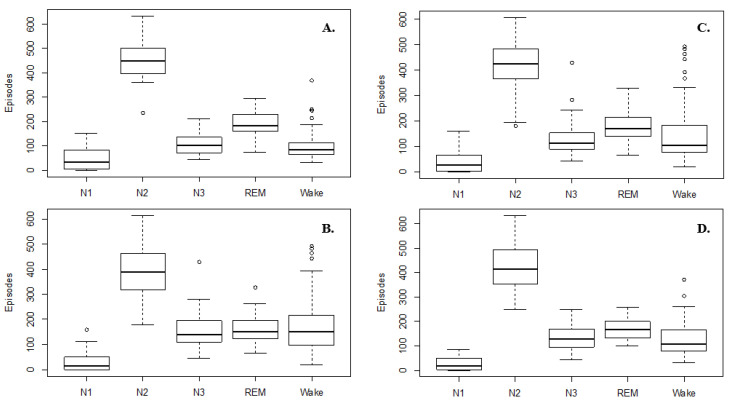
Distribution of sleep structure for (**A**) control group, (**B**) OSA group, (**C**) training set, and (**D**) test set, ° is represented outliers.

The sleep structure includes wake, N1, N2, N3, and REM stages with four–six repeated cycles at night [21]. As shown in Figure 2, the sleep structure’s distribution is different for the control and OSA groups; therefore, we randomly mixed the control and OSA groups to obtain a dataset with a similar sleep structure for the training (Figure 2C) and evaluation (Figure 2D) of the constructed DCR model.

### 2.3. DCR Model

Our constructed DCR model considers the characteristics of human sleep, such as its complex structure and cyclic rhythm. The convolutional networks in this model can extract high-dimensional feature maps from the input ECG signal to represent the complex structure of sleep, and the deep recurrent units play a role in capturing the cyclic rhythm of sleep. To construct the DCR model for the automatic scoring of sleep stages, a one-dimensional (1D) convolution and 1D pooling were used in the convolutional network, and a gated recurrent unit (GRU) contributed to the recurrent network. As an ECG signal is regarded as a time-series signal, a 1D convolution was used to implement the convolutional network. GRU is a robust technique for recurrent neural networks [22], which is why it was used in the constructed DCR model. To optimize the DCR model, batch normalization [23], dropout [24], and a rectified linear unit (ReLU) [25] were used with their appropriate settings through trial and error. These techniques are described below.

One-dimensional convolution: a one-dimensional convolution is suitable for application to physiological signals such as ECGs because it is simpler and faster than two-dimensional convolutions. A 1D convolution can be represented by the following expression:

(1) $$x_{k}=b_{k}+\sum_{i=1}^{N}w_{k}\times y_{i}$$

where *x_k_* is the *k*-th feature map, *b_k_* is the bias of the *k*-th feature map, *w_k_* is the *k*-th convolutional kernel from all features of the *k*-th feature map, and *y_i_* represents the *i*-th feature map.

One-dimensional pooling: pooling was used to reduce the dimensions of the intermediate feature maps. If a 1D kernel is used in the pooling operation, it is called 1D pooling. All pooling layers used max-pooling.

Gated recurrent unit: GRU, introduced by Chung et al. [22], is simpler and faster than LSTM. A GRU only has two gates: an update gate z and a reset gate r. The reset gates can capture short-term dependencies in sleep stages, whereas update gates capture long-term dependencies in sleep stages. The gating mechanism of GRU is expressed as follows:

(2) $$z_{t}=f(W_{z}\cdot [h_{t-1},x_{t}])$$

(3) $$r_{t}=f(W_{r}\cdot [h_{t-1},x_{t}])$$

(4) $${\tilde{h}}_{t}=\tanh(W\cdot [r_{t}*h_{t-1},x_{t}])$$

(5) $$h_{t}=(1-z_{t})*h_{t-1}+z_{t}*{\tilde{h}}_{t}$$

where *x_t_* is the mini-batch input at time step *t*, *h_t-1_* is the hidden state of the last time step, or the old state, *h_t_* is the new state, ${\tilde{h}}_{t}$ is the new candidate state, *W_z_* and *W_r_* are the respective weight parameters of the reset and update gates, and *f* is a sigmoid function.

Batch normalization: this was applied to the input ECG signal before training the constructed DCR model, as shown in Equation (6):

(6) $$x_{b}=\alpha \cdot \left(\frac{x_{i}-\mu }{\sqrt{\sigma^{2}+\varepsilon }}\right)+\beta$$

where *ε* is a small random noise, *μ* is the mini-batch mean, *σ* is the mini-batch variance, *α* denotes a scale parameter, and *β* represents a shift parameter. Both *α* and *β* are trainable and updated in an epoch-wise manner [23].

Dropout: this technique refers to randomly dropping out nodes in a network to reduce network model overfitting by preventing complex adaptations to training data [24].

ReLU: ReLU was used as the activation function in each layer of the DCR model, and can be represented as follows:

(7) f(x)=max(0,wx+b)

where *x* represents the feature map, *w* is the weight, and *b* is the bias. ReLU has shown a robust training performance and consistent gradients, thereby aiding gradient-based learning [25].

Structure of DCR model: Table 2 shows the detailed characteristics of the constructed DCR model’s final architecture. The DCR model has a five-layer structure, which includes a three-layer 1D convolution and a two-layer GRU.

In each convolutional layer, the kernel sizes are 50 × 1, 30 × 1, and 10 × 1 for the 1D convolution operation, followed by the 1D pooling with a 2 × 1 size. Each recurrent layer contains 20 and 10 hidden nodes in the GRU. Finally, we use a fully-connected multilayer perceptron with soft-max activation for the final discrimination of sleep stage scoring.

### 2.4. Implementation

The PSG data in this study was processed using MATLAB (R2018b). The DCR model was constructed using the Keras library with a TensorFlow backend [26]. A workstation with an Intel CPU (i9-9900X @3.5GHz) and NVIDIA GPU (GeForce RTX 2080 TI) was used for deep learning. Finally, a performance comparison of the models was performed, based on a model constructed by repeated learning with batch sizes of 128 and 2000 episodes.

### 2.5. Evaluation Index

The F-measure was used to evaluate the constructed DCR model based on CNN and GRU; it evaluates the correct classification of each class based on class equality. To find the F-measure, two evaluation measures, precision and recall, are combined. These are defined as follows:

(8) $$\mathrm{precision}=\frac{\mathrm{TP}}{\mathrm{TP}+\mathrm{FP}}$$

(9) $$\mathrm{recall}=\frac{\mathrm{TP}}{\mathrm{TP}+\mathrm{FN}}$$

where TP, FP, and FN are the true positives, false positives, and false negatives, respectively. They represent the number of events. The F1-score is based on the sample proportion of precision and recall as follows:

(10) $$F1=2\times \frac{\mathrm{precision}*\mathrm{recall}}{\mathrm{precision}+\mathrm{recall}}$$

## 3. Results

The results from the DCR model for automatic scoring of sleep stages using a raw single-lead ECG signal are shown in Table 3 and Table 4. The DCR model was evaluated using precision, recall, F1-score, and accuracy scores for three- and five-class sleep stage scoring. For the three-class evaluation, N1, N2, and N3 were integrated into the NREM stage.

In the case of three-class sleep stage scoring (Table 3), the DCR model showed a robust performance with an accuracy of 81% for the training set and 86% for the test set. In particular, the DCR model achieved the best performance in the NREM stage and the worst performance in the wake stage among the three groups; however, its performance in the REM stage was not very high, even though this stage had the largest number of events.

We obtained reasonable performances for five-class sleep staging using a raw single-lead ECG without any feature extraction (Table 4). The results showed accuracies of 73% and 74% for the training and test sets, respectively. In the five-class sleep staging, the DCR model showed a better performance in the REM stage than in the three-class sleep staging; however, the overall accuracy of the DCR model was relatively high, indicating that it is well adapted to the characteristics of human sleep.

## 4. Discussion

In this study, a DCR model was constructed for the automatic scoring of sleep stages based on a raw single-lead ECG signal. In the DCR model, convolutional and recurrent networks were combined to apply the characteristics of human sleep. We obtained a robust performance with an accuracy of 74.2% for five-class sleep stages and 86.4% for three-class sleep stages from the raw single-lead ECG signal. In addition, the DCR model was evaluated using an ECG dataset obtained from the control and OSA groups.

Table 5 compares and analyzes existing studies that include an ECG signal or detect sleep stage scoring based on the features obtained from HRV and RR intervals. Several studies have dealt with three-class classification based on machine learning using a single-lead ECG signal. Most of these studies used a shallow learning classifier as a SVM over deep learning models, along with several handcrafted features extracted from ECG signals for a multiclass (wake, NREM, and REM) classification; however, the proposed three-class DCR model outperformed all these studies because it applied larger datasets and sequential sleep structures. Ebrahimi et al. [27] performed a multiclass classification including wake, stage 2, slow wave sleep, and REM, based on SVM, using a combination of HRV and thoracic respiratory signal features, and they achieved a good performance with a total accuracy of 89.32% for the test set when using 27 of the best features; however, they used two vital signs, HR and respiratory, along with long-duration episodes to extract these features.

Some studies based on the deep learning framework for automatic scoring of sleep stages used an ECG signal [17,18,19,28]. In these studies, deep learning frameworks are used instead of conventional classifiers or feature extractors. For example, Wei et al. [17] used a deep learning framework as a classifier and designed a four-layer DNN model for sleep stage classification based on a single-lead ECG signal. First, they detected QRS complexes and extracted RR intervals from a single-lead ECG signal to extract handcrafted features. Then, the features were extracted through a domain transformation analysis from the RR interval. Finally, they trained the designed DNN model using the top features to classify the sleep stages. In addition, LSTM was used as a classifier in a study by Radha et al. [19]. Before training the LSTM model, 135 features were extracted from the HRV signal to achieve an accuracy of 72.9% for five-class sleep stages. In contrast, Li et al. [18] used a CNN model as a feature extractor. Initially, they extracted vital signs from the ECG signal, including HRV and EDR, and then obtained the cardiorespiratory coupling data for feature extraction using the CNN model. CNN features and some nonlinear features were applied to the SVM classifier for the final discrimination.

In a study by Zhang et al. [28], the RNN model for sleep stage classification used HR and actigraphy from wearable devices. The HR was extracted from the PPG signal, and a transfer learned RNN model was used; however, its performance was the lowest among the studies listed in Table 5. Moreover, deep learning frameworks (DNN, CNN, RNN, and LSTM) have been used as feature extractors or classifiers in previous studies based on ECG signals; however, they have achieved a lower performance in five- or three-class sleep scorings.

Therefore, we constructed a DCR model that can proceed with feature extraction and classification sequentially from a raw ECG signal for automatic sleep staging. The constructed DCR model performs better than the conventional methods because it considers the complex and cyclic characteristics of sleep. Another advantage of the DCR model for sleep stage scoring is that it does not require intermediate vital signs (HRV, RR interval, and EDR) or any handcrafted feature sets extracted using a domain transformation analysis. In addition, the DCR model has a simpler structure than other deep learning models designed for sleep stage scoring, and it has been trained and tested on a clinical dataset consisting of control and OSA patient groups. Our results show the constructed DCR model’s reasonable multiclass classification performance for five- or three-class sleep stages using only a single-lead ECG signal. In addition, the DCR model can help as an alternative tool for sleep screening, monitoring, and healthcare apps and solutions.

This study has some limitations. As our study population is relatively small, the study findings should be clinically validated in a more diverse and larger study population. In addition, the proposed DCR model should be validated externally and publicly, using accessible data sets including Sleep Heart Health Study (SHHS) and the Computing in Cardiology Challenge. Subjects with any cardiovascular diseases or severe sleep apnea were excluded from this study. The proposed DCR model may not perform as expected for these subject groups. Finally, the DCR model needs more computational power than previous studies. In a further study, we will try to find a solution to the shortcomings of this study.

## 5. Conclusions

In summary, a DCR model is constructed for the automatic detection of sleep stages based on a raw single-lead ECG signal. Most ECG-based studies for sleep stage scoring were classified into binary or triple classes, but the DCR model can perform a multiclass classification for three- or five-class sleep stages. In addition, the DCR model can automatically extract the feature maps and classify the sleep stages at once from the raw ECG signal. We obtained a high performance with an overall accuracy of 86% for three-class sleep stages and 74% for five-class sleep stages; therefore, a DCR model can be appropriate for sleep stage scoring from a raw single-lead ECG signal without any feature extraction. Furthermore, a validation study should be conducted on the DCR model, which uses larger and more diverse datasets based on a single-lead ECG signal.

## Figures and Tables

**Table 1:** Demographics of the study population.

Dataset	Measures	Control	OSA	Total
All subjects	*N* (M:F)	52 (25:27)	60 (46:14)	112 (71:41)
	Age (years)	48.0 ± 5.8	58.4 ± 11.2	53.5 ± 10.5
	BMI (kg/m^2^)	22.6 ± 1.8	25.6 ± 3.1	24.2 ± 3.0
	AHI (per hour)	2.3 ± 2.3	17.5 ± 6.8	10.4 ± 9.3
	TST (hour)	6.5 ± 0.8	5.9 ± 0.9	6.2 ± 0.9
	SE (%)	90.1 ± 11.0	80.7 ± 11.8	85.1 ± 12.3
Training set	*N* (M:F)	42 (18:24)	47 (37:10)	89 (55:34)
	Age (years)	48.0 ± 6.0	57.9 ± 11.7	53.1 ± 10.5
	BMI (kg/m^2^)	22.6 ± 1.7	25.7 ± 3.2	24.2 ± 3.0
	AHI (per hour)	2.3 ± 2.4	18.0 ± 7.0	10.6 ± 9.5
	TST (hour)	6.5 ± 0.9	5.9 ± 1.0	6.2 ± 1.0
	SE (%)	89.7 ± 12.1	80.5 ± 12.5	84.8 ± 13.1
Test set	*N* (M:F)	10 (7:3)	13 (9:4)	23 (16:7)
	Age (years)	48.2 ± 7.5	60.5 ± 9.2	55.1 ± 10.4
	BMI (kg/m^2^)	22.9 ± 2.4	25.2 ± 3.2	24.2 ± 3.0
	AHI (per hour)	2.3 ± 2.1	15.9 ± 5.6	10.0 ± 8.5
	TST (hour)	6.5 ± 0.4	6.0 ± 0.7	6.2 ± 0.7
	SE (%)	91.9 ± 3.8	81.4 ± 8.8	86.0 ± 8.7

Note: BMI, body mass index; AHI, apnea hypopnea index; TST, total sleep time; SE, sleep efficiency.

**Table 2:** Detailed architecture of the constructed DCR model.

Layers	Filters (Kernel Size)	Output Shape	Parameters
Batchnorm_1	=	3000 × 1	4
Conv1d_1	60 (50 × 1)	2951 × 60	3060
Maxpool1d_1	2 × 1	1475 × 60	
Dropout_1	*p* = 0.25		
Conv1d_1	30 (30 × 1)	1446 × 30	54,030
Maxpool1d_1	2 × 1	723 × 30	
Dropout_1	*p* = 0.25		
Conv1d_1	10 (20 × 1)	704 × 10	6010
Maxpool1d_1	2 × 1	352 × 10	
Dropout_1	*p* = 0.25		
GRU_1	20	352 × 20	1920
Dropout_4	*p* = 0.25		
GRU_2	10	352 × 10	960
Dropout_5	*p* = 0.25		
Fullyconn_1	3 5	10 × 3 10 × 5	33 55
3 CNN, 2 GRU	Totally 130 filters and 66,015 parameters		

**Table 3:** Performance for three-class sleep staging in the test set.

Dataset	Sleep Stages	Precision	Recall	F1-Score	Accuracy
Training set	Wake	0.78	0.58	0.66	0.87
	REM	0.95	0.89	0.92	
	NREM	0.62	0.84	0.72	
Test set	Wake	0.86	0.68	0.76	0.86
	REM	0.92	0.87	0.89	
	NREM	0.56	0.82	0.71	

**Table 4:** Performance for five-class sleep staging in the test set.

Dataset	Sleep Stages	Precision	Recall	F1-Score	Accuracy
Training set	Wake	0.63	0.66	0.65	0.77
	REM	0.77	0.93	0.84	
	N1	0.51	0.16	0.24	
	N2	0.83	0.76	0.79	
	N3	0.79	0.73	0.76	
Test set	Wake	0.59	0.64	0.62	0.74
	REM	0.75	0.91	0.82	
	N1	0.39	0.14	0.20	
	N2	0.79	0.71	0.74	
	N3	0.75	0.66	0.70	

**Table 5:** Comparison of ECG-based sleep stage scoring studies.

Author	Signal	Method	Classes	Accuracy
Adnane et al. [13]	HRV	SVM	2	79.9
Xiao et al. [14]	HRV	RF	3	72.5
Ebrahimi et al. [27]	HRV, Resp.	SVM	4	89.3
Singh et al. [15]	RR interval	SVM	2	72.8
Yücelbaş et al. [16]	ECG	RF	3	78.0
Wei et al. [17]	ECG	DNN	3	77.8
Li et al. [18]	ECG→CRC	CNN	3	73.0
Radha et al. [19]	HRV,	LSTM	5	72.9
Zhang et al. [28]	HR, Actigraphy	RNN	3	66.6
This study	ECG	DCR	5	74.2
			3	86.4

## Data Availability

Not applicable.
